# A STRIPAK component Strip regulates neuronal morphogenesis by affecting microtubule stability

**DOI:** 10.1038/srep17769

**Published:** 2015-12-08

**Authors:** Chisako Sakuma, Misako Okumura, Tomoki Umehara, Masayuki Miura, Takahiro Chihara

**Affiliations:** 1 Department of Genetics, Graduate School of Pharmaceutical Sciences, The University of Tokyo; 2 AMED-CREST, JST, 7-3-1 Hongo, Bunkyo-ku, Tokyo 113-0033, Japan

## Abstract

During neural development, regulation of microtubule stability is essential for proper morphogenesis of neurons. Recently, the striatin-interacting phosphatase and kinase (STRIPAK) complex was revealed to be involved in diverse cellular processes. However, there is little evidence that STRIPAK components regulate microtubule dynamics, especially *in vivo*. Here, we show that one of the core STRIPAK components, Strip, is required for microtubule organization during neuronal morphogenesis. Knockdown of Strip causes a decrease in the level of acetylated *α*-tubulin in *Drosophila* S2 cells, suggesting that Strip influences the stability of microtubules. We also found that Strip physically and genetically interacts with tubulin folding cofactor D (TBCD), an essential regulator of *α*- and *β*-tubulin heterodimers. Furthermore, we demonstrate the genetic interaction between *strip* and *Down syndrome cell adhesion molecule* (*Dscam*), a cell surface molecule that is known to work with TBCD. Thus, we propose that Strip regulates neuronal morphogenesis by affecting microtubule stability.

Microtubules are crucial building blocks of axons and dendrites. They provide physical support, establish polarization, and serve as ‘tracks’ for intracellular transport that enable trafficking of various molecules between cell body and neurite tips. However, microtubules are intrinsically dynamic, which means that the microtubule cytoskeleton can rapidly rearrange in response to internal or external cues. Various molecules support microtubule dynamics, which involves the proper regulation of polymerization and depolymerization of microtubules. Numerous microtubule-associated proteins (MAPs) including structural MAPs, plus-end tracking proteins (+TIPs), and motor proteins directly regulate microtubule assembly and depolymerization^1^,^2^,^3^. Furthermore, post-translational modifications of microtubules are also important for their dynamics by forming a biochemical ‘tubulin code’ that can be ‘read’ by microtubule-interacting factors^4^. For example, acetylation of *α*-tubulin influences the degree of kinsesin-1 interaction with the microtubules^5^. Acetylation of *α*-tubulin also influences microtubule sensitivity to be severed by katanin^6^, another mode of regulation of microtubule dynamics that is particularly important for branch formation^7^. Thus, the stringently regulated microtubule dynamics are crucial for both development and maintenance of neurites, and many neuronal diseases are linked to microtubules^8^.

The striatin-interacting phosphatase and kinase (STRIPAK) complex is an evolutionarily conserved complex that is recently revealed to have roles in various cellular processes including, signaling, cell cycle control, apoptosis, vesicular trafficking, Golgi assembly, cell polarity and cell migration^9^. Moreover, STRIPAK complexes have been linked to clinical conditions, including cardiac disease, diabetes, autism, and cerebral cavernous malformation. The core of the STRIPAK complex is the striatin family of proteins that serve as B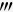 subunits (one of the subfamily of regulatory B subunits) of protein phosphatase 2A (PP2A) complex. In addition to Striatins, A and C subunits of PP2A, Mob3, Mst3, Mst4, Ysk1, Ccm3, and Strip1 and 2 are known to form the core mammalian STRIPAK complex^10^,^11^. This core complex binds additional proteins in a mutually exclusive manner to form distinct STRIPAK complexes involved in diverse functions^9^. Although our knowledge of the composition of the striatin family complexes has increased greatly, much remains to be determined regarding the function of these complexes and the roles of the various striatin family-associated proteins^9^. There have been some indications that STRIPAK complexes might affect microtubule organization. For example, dMob4, the *Drosophila* Mob3 homolog is required for microtubule morphology at neuromuscular junctions, peripheral nerves and muscles^12^. Furthermore, STRIP2 knockdown in PC3 prostate cancer cells altered microtubule organization and induced cell elongation^13^. It was also reported that Dynein, a minus-end directed motor, associates with the STRIPAK complex using an affinity-purification mass spectrometry analysis^10^. However, the precise molecular mechanism of microtubule regulation by STRIPAK complexes *in vivo* is unclear.

We previously demonstrated that *Drosophila* Strip, the homolog of mammalian Strip1 and 2, regulates dendrite branching and axon elongation in *Drosophila* olfactory projection neurons^14^. We revealed that Strip serves as a platform for early endosome organization during axon elongation. The shorter axon phenotype caused by *strip* knockdown was suppressed by the expression of constitutive active form of Rab5, one of the key regulators of early endosome fusion. However, the suppression was partial, and the dendrite overbranching phenotype was not suppressed. We hypothesized that Strip might also form complexes with other molecules that affect axon elongation and dendrite branching. Thus, we speculated that Strip might affect microtubule organization, since microtubules are crucial components of axons and dendrites, and microtubule dynamics should be strictly regulated during neural development.

Here we show that Strip forms a complex with microtubules and affects their stabilization. Furthermore, we reveal that *strip* genetically interacts with *tubulin folding cofactor D* (*TBCD*), one of the tubulin-folding cofactors that assist the tubulin heterodimer formation and control the availability of tubulin subunits and microtubule stability^15^, to regulate neuronal morphogenesis. Moreover, we show that Strip cooperates with Down syndrome cell adhesion molecule (Dscam), a cell surface molecule whose intracellular domain binds to TBCD and it is likely that TBCD mediates Dscam functions by affecting microtubule dynamics^16^.

## Results

### Strip is localized along microtubules

**Figure 1:**
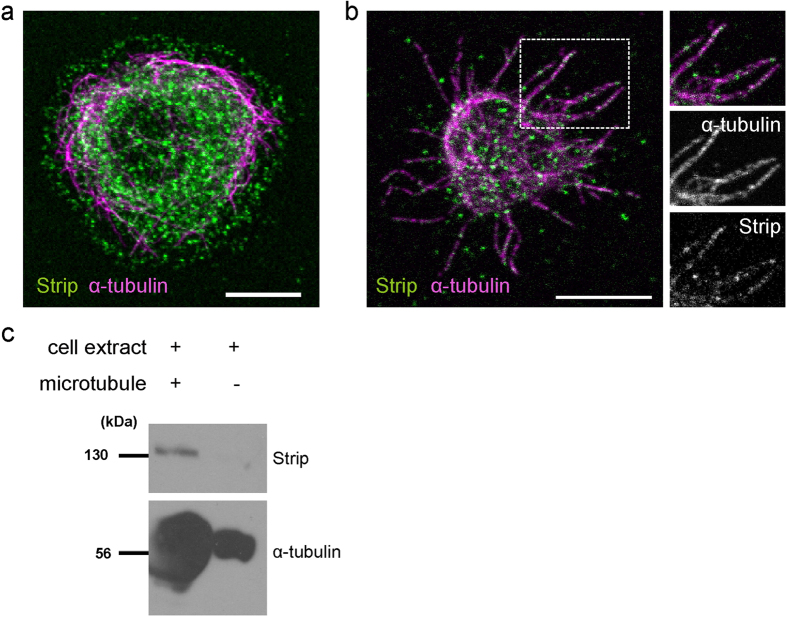
Strip interacts with microtubule. (**a**) *Drosophila* S2 cells plated on a concanavalin-A coated cover slip were immunostained with anti-Strip (green) and anti-*α*-tubulin (magenta) antibodies. Scale bar is 7.5 μm. (**b**) S2 cells were treated with the extraction method for clear observation of microtubules. S2 cells are immunostained with anti-Strip (green) and anti-*α*-tubulin (magenta) antibodies. Dotted rectangle area is enlarged in the right panels. Scale bar is 7.5 μm. (**c**) S2 cell extract was incubated with *in vitro*-polymerized microtubules or buffer only and pelleted by centrifugation. The pellet was denatured and subjected to western blotting using anti-Strip and anti-*α*-tubulin antibodies.

To understand the relationship between Strip and microtubule, we first examined the subcellular localization of Strip in *Drosophila* S2 cells. It is difficult to evaluate whether Strip is localized on microtubules because endogenous Strip seems to be distributed throughout the cytoplasm (Fig. 1a). Therefore, we utilized the extraction method^17^ to remove the cytosolic components and visualize cytoskeleton-associated proteins. As we expected, Strip was localized along microtubules (Fig. 1b). To further investigate this relationship, we performed the microtubule co-sedimentation assay. When *in vitro*-polymerized microtubules with no Strip were incubated with the extracts of S2 cells and pelleted by centrifugation through a glycerol cushion, S2 cell-derived Strip was associated with the microtubules (Fig. 1c). These data demonstrate that Strip forms a complex with microtubules.

### Strip affects organization and stability of microtubule

**Figure 2:**
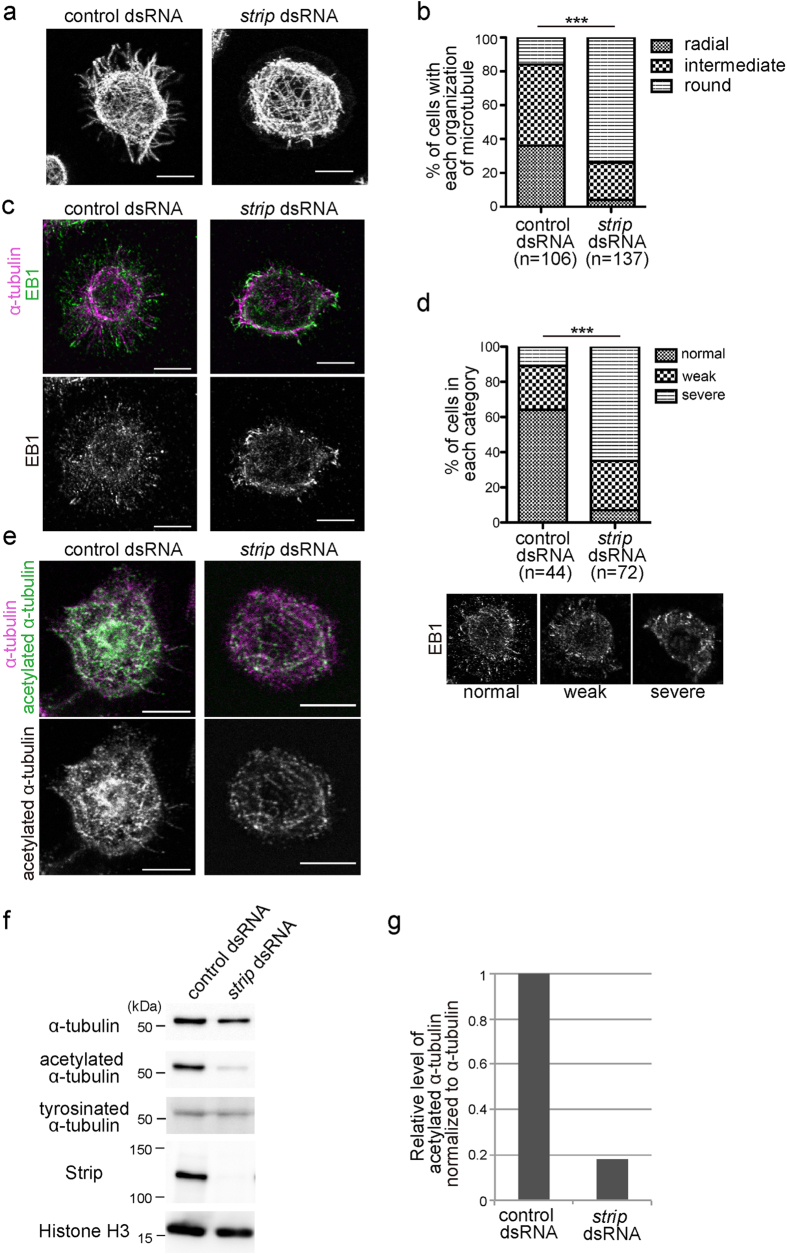
*strip* knockdown affected microtubule stability. (**a**) S2 cells were treated with control or *strip* dsRNA for 8 days and immunostained with anti-*α*-tubulin antibody. Scale bar is 7.5 μm. (**b**) All S2 cells are classified into having radial projections to cell periphery, not having radial projection (overall microtubule morphology was rounded) or intermediate by a blind test. ***P < 0.0001, chi-square test. (**c**) S2 cells were treated with control or *strip* dsRNA for 8 days and immunostained with anti-EB1 (green) and anti-*α*-tubulin (magenta) antibodies. (**d**) All cells were blindly classified into three categories, scattered and small EB1 comets (normal), intermediate (weak), fewer comets*/*irregular EB1 accumulation (severe). ***P < 0.0001, chi-square test. Scale bar is 7.5 μm. (**e**) S2 cells were treated with control or *strip* dsRNA for 8 days and immunostained with anti-acetylated-*α*-tubulin (green) and anti-*α*-tubulin (magenta) antibodies. Scale bar is 7.5 μm. (**f**) Immunoblots of lysate of S2 cells treated with control or *strip* dsRNA for 8 days. Since Strip seems to slightly affect the total *α*-tubulin level, Histone H3 is used as a loading control. (**g**) Densitometric analysis of acetylated-*α*-tubulin. The level of acetylated-*α*-tubulin is normalized to *α*-tubulin. This reduction of the level of acetylated-*α*-tubulin in *strip* dsRNA-treated cells was confirmed for 10 times.

We next examined the role of Strip on microtubule by treating S2 cells with *strip* dsRNA. Most of the microtubules showed radial projections from the cell center to the periphery in control dsRNA-treated S2 cells (Fig. 2a,b). In contrast, radial projections towards cell periphery were mostly absent and overall microtubule morphology was rounded in *strip* dsRNA-treated S2 cells (Fig. 2a,b). We further investigated the localization of microtubule plus end tracking protein, EB1 to monitor the microtubule organization and found that EB1 distribution was obviously altered in *strip* dsRNA-treated S2 cells (Fig. 2c,d). EB1 was localized at the periphery of wild-type S2 cells in a small comet-like pattern while the number of EB1 comets was significantly reduced and sometime irregular EB1 accumulations were observed in *strip* dsRNA-treated S2 cells.

Multiple post-translational modifications in tubulins are crucial for dynamics and organization of microtubules^18^ and can be used to monitor different populations of microtubules. Therefore, we tried to investigate the level of acetylated and tyrosinated *α*-tubulin in *strip* dsRNA-treated S2 cells. Acetylated *α*-tubulins exist in stable, long-lived microtubules that are resistant to nocodazole^19^,^20^. Tyrosinated *α*-tubulins are found in newly formed microtubules since tubulin detyrosination occurs after the incorporation of tubulin subunits into the microtubule lattice^21^. Interestingly, the level of acetylated *α*-tubulin was greatly reduced when cells were treated with *strip* dsRNA, indicating that microtubule stability was decreased when *strip* was knocked down (Fig. 2e–g). The level of tyrosinated tubulin was not drastically changed in *strip* dsRNA-treated cells (Fig. 2f).

### *strip* and *tubulin folding cofactor D* cooperatively regulate neuronal morphogenesis

**Figure 3:**
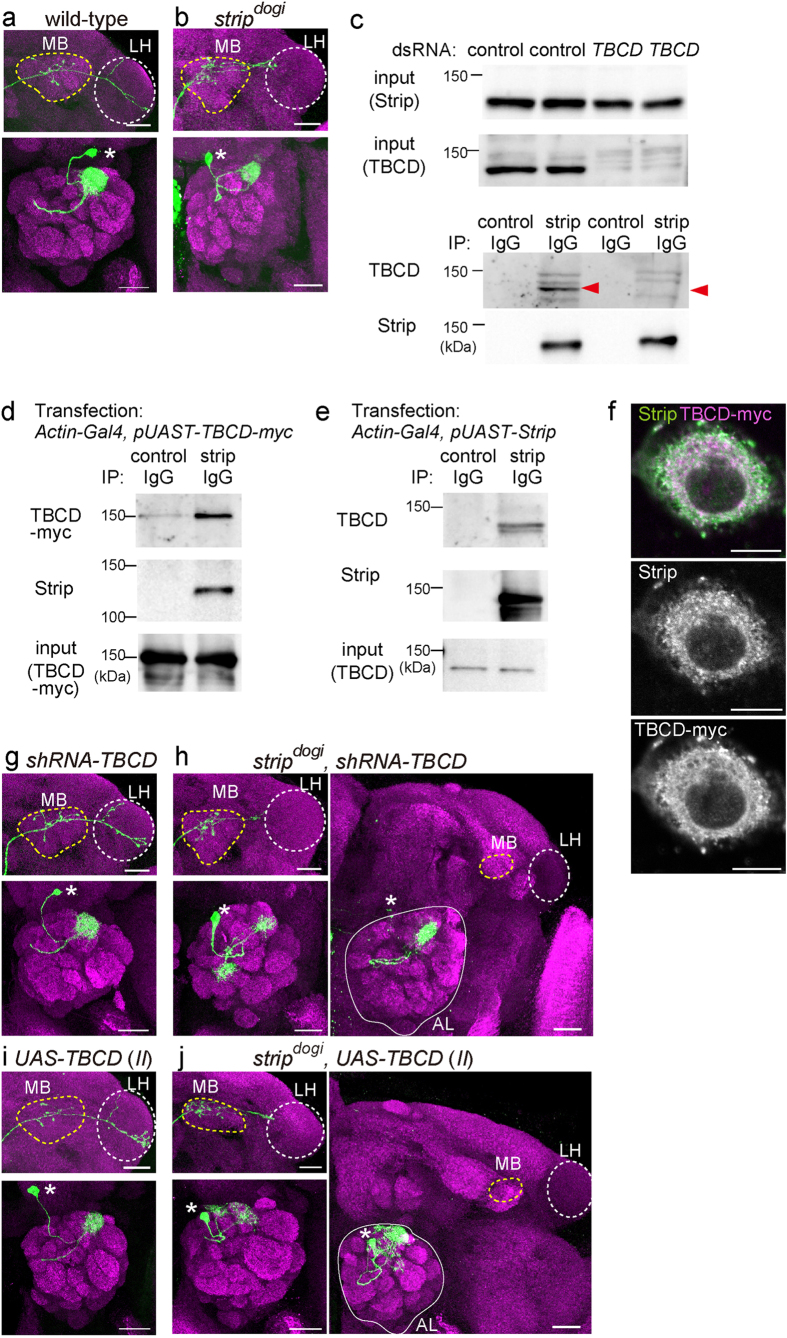
Strip physically and genetically interacts with TBCD. (**a**,**b**) Representative images of wild-type (**a**) or *strip*^*dogi*^ (**b**) DL1 PN clones (green). Bruchpilot (Brp) staining in magenta. Scale bar is 25 μm. Asterisk: cell body. Yellow and white dotted circles indicate the mushroom body (MB) and the lateral horn (LH), respectively. (**c**) Immunoprecipitation of S2 cell lysates treated with control or *TBCD* dsRNA for 6 days. Endogenous TBCD was co-immunoprecipitated when endogenous Strip was precipitated by anti-Strip antibody in control dsRNA-treated S2 cells but not in *TBCD* dsRNA-treated cells. Red triangles indicate endogenous TBCD. (**d**) Immunoprecipitation of S2 cell lysates expressing c-myc tagged TBCD (TBCD-myc). TBCD-myc was co-immunoprecipitated when endogenous Strip was precipitated by anti-Strip antibody. (**e**) Immunoprecipitation of S2 lysates expressing Strip. Endogenous TBCD was co-immunoprecipitated when Strip was precipitated by anti-Strip antibody. (**f**) Representative image of S2 cells expressing TBCD-myc. Magenta: TBCD-myc, green: endogenous Strip. (**g**,**h**) Representative images of single-cell clones of DL1 PNs expressing shRNA against *TBCD* (*shRNA-TBCD*) in wild type (**g**) or *strip*^*dogi*^ (**h**) PNs. (**i**,**j**) Representative images of single-cell clones of DL1 PNs overexpressing *TBCD* in wild type (**i**) or *strip*^*dogi*^ (**j**) PNs. The right panels in (**h**) and (**j**) show PNs exhibiting the ‘extremely short axon’ phenotype, i.e., the axon did not exit the antennal lobe (white circle) and did not enter the mushroom body (MB, yellow dotted circle) or the lateral horn (LH, white dotted circle). Scale bar is 25 μm. Genotypes: (**a**) *y w, hs-FLP122, UAS-mCD8GFP/y w* (*or Y*)*; GH146-Gal4, UAS-mCD8-GFP/+; tubP-Gal80, FRT*^2A^*/FRT*^*2A*^, (b) *y w, hs-FLP122, UAS-mCD8GFP/y w* (*or Y*)*; GH146-Gal4, UAS-mCD8-GFP/+; tubP-Gal80, FRT*^*2A*^*/strip*^*dogi*^*, FRT*^*2A*^*, y+*, (g) *y w, hs-FLP122, UAS-mCD8GFP/y w* (*or Y*)*; GH146-Gal4, UAS-mCD8-GFP/UAS-shRNA-TBCD*^*#1*^*; tubP-Gal80, FRT*^*2A*^*/FRT*^*2A*^, (**h**) *y w, hs-FLP122, UAS-mCD8GFP/y w* (*or Y*)*; GH146-Gal4, UAS-mCD8-GFP/UAS-shRNA-TBCD*^*#1*^*; tubP-Gal80, FRT*^*2A*^*/strip*^*dogi*^*, FRT*^*2A*^*, y+*, (**i**) *y w, hs-FLP122, UAS-mCD8GFP/y w* (*or Y*)*; GH146-Gal4, UAS-mCD8-GFP/UAS-TBCD; tubP-Gal80, FRT*^*2A*^*/FRT*^*2A*^, (**j**) *y w, hs-FLP122, UAS-mCD8GFP/y w* (*or Y*)*; GH146-Gal4, UAS-mCD8-GFP/UAS-TBCD; tubP-Gal80, FRT*^*2A*^*/strip*^*dogi*^*, FRT*^*2A*^*, y+*.

To investigate whether Strip also influences the stability of microtubules *in vivo*, we performed a mosaic analysis with a repressible cell marker (MARCM)-based analysis^22^ in *Drosophila* olfactory projection neurons (PNs), an excellent model system for studying the molecular mechanisms of neuronal morphogenesis^23^,^24^,^25^. We analyzed MARCM single-cell clones of PNs whose dendrites target the DL1 glomerulus in the antennal lobe (DL1 PNs, Fig. 3a). Axons of the DL1 PNs target the mushroom body and the lateral horn where the axon exhibits stereotypical L-shape branching (Fig. 3a). As we reported previously, single-cell clones of PNs homozygous for *strip*^*dogi*^ (hereafter, *strip*^*dogi*^ PN) show defects in axon elongation and dendrite branching^14^. In *strip*^*dogi*^ PNs, an additional dendrite branch appeared from the proximal side of the dendrite shaft (overbranching phenotype: 10.26%, n = 4/39, Fig. 3b) and two-glomerular-targeting dendrites were also observed at a low frequency^14^. The axon of *strip*^*dogi*^ PNs did not elongate or form L-shaped branches in the lateral horn (97.2%, n = 35/36, Fig. 3b).

To determine whether Strip is involved in microtubule dynamics during neuronal morphogenesis, we examined the relationship between *strip* and *TBCD*. TBCD is one of the five tubulin-folding cofactors and assists in the formation of tubulin heterodimers^26^. Recently, we reported that an optimum level of TBCD is crucial for neuronal morphogenesis. Interestingly, PNs homozygous for *TBCD*^*1*^ mutation or overexpressing *TBCD* show similar phenotypes with *strip*^*dogi*^ PNs; both PNs exhibit dendrite overbranching phenotype^16^. Furthermore, *TBCD*^*1*^ PNs exhibits shorter axon phenotype as *strip*^*dogi*^ PNs. Based on these phenotypic similarities between *strip* and *TBCD* mutant, we hypothesized that Strip could function with TBCD to regulate neuronal morphogenesis. First, we examined the physical interaction between Strip and TBCD by performing a co-immunoprecipitation assay using S2 cell lysate, and found that Strip and TBCD can form a protein complex (Fig. 3c–e). Next, we examined the subcellular localization of Strip and TBCD-myc in S2 cells and found that endogenous Strip and TBCD-myc are mostly co-localized (Fig. 3f). We then examined the genetic interaction between *strip* and *TBCD* by expressing short-hairpin RNA for TBCD (*shRNA-TBCD)* or overexpressing *TBCD* in *strip*^*dogi*^ PNs. Although PNs homozygous for *TBCD*^*1*^ mutation exhibited defects in dendrite branching and axon elongation, *shRNA-TBCD* expression in DL1 PNs did not cause obvious phenotypes either in dendrites or axons, probably owing to its weak knockdown efficiency (Fig. 3g). In addition, *TBCD* overexpression also did not cause obvious phenotype (Fig. 3i), which was different from our previous report^16^ that used different *UAS-TBCD* transgenic line (See Methods for more details). When *shRNA-TBCD* or *TBCD* was expressed in *strip*^*dogi*^ PNs, the phenotypes of *strip*^*dogi*^ PNs were significantly enhanced. The penetrance of two-glomeruli-targeting dendrites was increased (2.56%, n = 1/39 for *strip*^*dogi*^ PNs, Fig. 3b; 37.5% n = 9/24 for *shRNA-TBCD* expressing *strip*^*dogi*^ PNs, Fig. 3h; 30% n = 3/10 for *TBCD* expressing *strip*^*dogi*^ PNs, Fig. 3j), and some axons showed ‘extremely short axon’ phenotype, that is, the axons did not exit the antennal lobe; this was never observed in *strip*^*dogi*^PNs (9.52%, n = 2/21, for *shRNA-TBCD* expressing *strip*^*dogi*^ PNs, Fig. 3h; 20%, n = 2/10, for *TBCD* expressing *strip*^*dogi*^ PNs, Fig. 3j). These results suggest that Strip interacts with TBCD during neuronal morphogenesis of PNs.

### Strip cooperates with Dscam in mushroom body neurons

**Figure 4:**
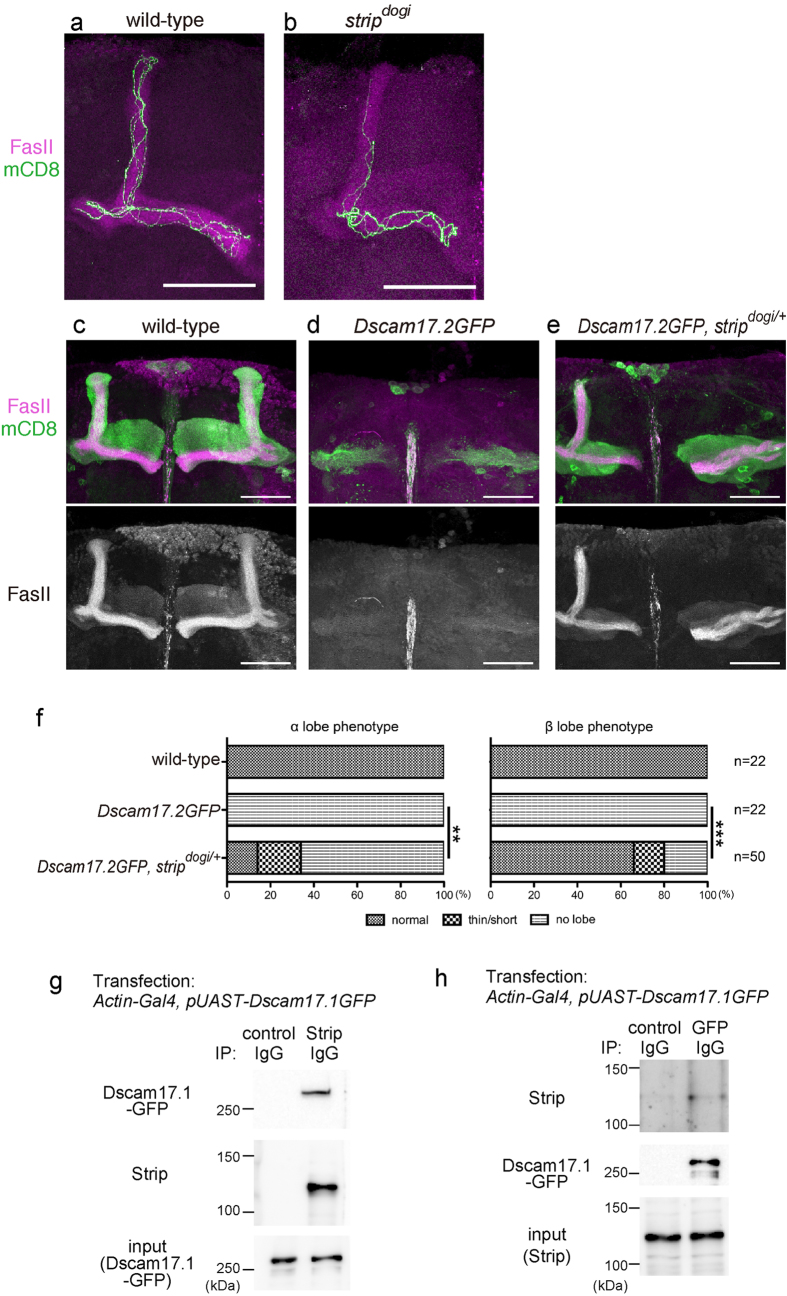
Strip cooperates with Dscam in the morphogenesis of mushroom body neurons. (**a**,**b**) Representative images of control (**a**) or *strip*^*dogi*^ (**b**, 11.8%, n = 8/68) homozygous clones of mushroom body *α*/*β* neurons. *UAS-mCD8-GFP* (green) is expressed by *OK107-Gal4*. Anti-FasII antibody was used to visualize MB *α*/*β* lobes (magenta). Scale bar is 50 μm. (**c**–**e**) Representative images of mushroom body neurons for each genotype. *UAS-mCD8-GFP* (green) is expressed by *OK107-Gal4*. Anti-FasII antibody was used to visualize MB *α*/*β* lobes (magenta). Scale bar is 50 μm. (**f**) Quantification of phenotypes of *α* and *β* lobes. **P = 0.0075, ***P < 0.0001, chi-square test. (**g**,**h**) Immunoprecipitation of S2 lysates expressing Dscam17.1-GFP. Dscam17.1-GFP was co-immunoprecipitated when Strip was precipitated by anti-Strip antibody (**g**). Endogenous Strip was co-immunoprecipitated when Dscam17.1-GFP was precipitated by anti-GFP antibody (**h**). Genotypes: (**a**) *y w, hs-flp, UAS-mCD8GFP/y w (or Y); UAS-βtub56D-myc/+; tub-Gal80, FRT*^*2A*^*/FRT*^*2A*^*, y(+); OK107-Gal4/+* (**b**) *y w, hs-flp, UAS-mCD8GFP/y w (or Y); UAS-βtub56D-myc/+; tub-Gal80, FRT*^*2A*^*/strip*^*dogi*^*, FRT*^*2A*^*, y(+); OK107-Gal4/+* (**c**) *y w, hs-FLP, UAS-mCD8GFP/w* (*or Y*)*; +/Cy, y+; OK107-Gal4/+*, (**d**) *y w, hs-FLP, UAS-mCD8GFP/y w* (*or Y*)*; Dscam17.2GFP/Cy, y+;; OK107-Gal4/+*, (**e**) *y w, hs-FLP, UAS-mCD8GFP/y w* (*or Y*)*;; strip*^*dogi*^*, FRT*^*2A*^*, y+/TM3, Sb; OK107-Gal4/+*.

As we reported previously, TBCD forms complex with Dscam and cooperates to regulate morphology of mushroom body neurons^16^. The mushroom body is an olfactory learning and memory centre^27^ and consists of three types of neurons, *α*/*β*, *α*′/*β*′, and γ neurons^28^. *α*/*β* neurons extend one axon into the *α* lobe and the other axon to the *β* lobe, that are labelled by anti-FasII antibody (Fig. 4a). Here we hypothesized that Strip, like TBCD, also acts in the downstream of Dscam. To test this hypothesis, we first generated MARCM clones of mushroom body *α*/*β* neurons homozygous for *strip*^*dogi*^(*strip*^*dogi*^mushroom body neurons). We found that *strip*^*dogi*^mushroom body neurons exhibited axon segregation phenotypes (Fig. 4b; 11.8% (n = 8/68) of *strip*^*dogi*^ clones exhibited less axons into the α lobes) that were similar to those in *Dscam* mutant mushroom body neurons^29^,^30^. We further provide the genetic evidence that *Dscam* and *strip* interact in mushroom body neurons. The ‘*α* and *β* lobes missing’ phenotypes were observed when Dscam was overexpressed in the mushroom body neurons (Fig. 4c,d,f), which was suppressed in the *TBCD*^*1*^ heterozygous background^16^. Similarly, *strip*^*dogi*^heterozygous background could also suppress ‘*α* and *β* lobes missing’ phenotype (Fig. 4e,f). Next we performed co-immunoprecipitation experiment. Anti-Strip antibody could co-immunoprecipitated endogenous Strip with Dscam-GFP (Fig. 4g). We could further confirm this interaction by immunoprecipitating Dscam-GFP by anti-GFP antibody; endogenous Strip was co-immunoprecipitated with Dscam-GFP (Fig. 4h). From these results, we concluded that Strip cooperates with Dscam in mushroom body neurons, possibly through interacting with TBCD.

## Discussion

To form a functional neural circuit, the morphology of each neuron should be strictly regulated to wire with the correct synaptic partners. Microtubules are fundamental structural components of axons and dendrites and their dynamics directly affect neuronal morphology. In this report, we show that Strip, one component of the STRIPAK complex, affects microtubule stability in *Drosophila* S2 cells. Furthermore, we report that Strip regulates dendrite branching and axon elongation by interacting with TBCD in the olfactory projection neurons. Moreover, we observed genetic interactions between *strip* and *Dscam* in the mushroom body neurons, suggesting that Strip and TBCD regulate microtubule stability in the downstream of Dscam.

We recently found that TBCD can bind to the intracellular domain of Dscam and it is likely that TBCD mediates Dscam functions by affecting microtubule dynamics^16^. Although Dscam has been extensively studied, the downstream pathways are not well known. The Dock/Pak signaling pathway, one of the known pathways acting downstream of Dscam, does not seem to be required for dendrite targeting and axon guidance of olfactory projection neurons^31^. Strip seems to act with TBCD in the downstream of Dscam during neuronal morphogenesis, and link outer cellular environment with microtubules.

*strip* and *TBCD* mutants show similar phenotypes in axon elongation and dendrite branching, and Strip and TBCD genetically and physically interact with each other. TBCD assists in the formation of tubulin heterodimers, and affects microtubule stability by controlling the availability of tubulin subunits because concentration of free tubulin dimers can affect microtubule dynamics^15^. Although mammalian TBCD does not bind to microtubules^32^, Alp1, a homolog of TBCD in *Schizosaccharomyces pombe*, is colocalized with microtubules^33^. Furthermore, overproduction of Cin1p, the homolog of TBCD in *Saccharomyces cerevisiae*, resulted in an increased sensitivity to benomyl^34^, an antifungal compound that weakly inhibits microtubule assembly^35^. Thus, TBCD and its homologs have been shown to regulate microtubule dynamics.

Strip seems to affect microtubule stability by interacting with several molecules in addition to TBCD. We previously reported that Strip also forms a complex with Glued, the homolog of mammalian p150^Glued,^^14^. Glued is one of the components of the dynactin complex required for dynein motor-mediated retrograde transport along microtubules. Glued has the CAP-Gly domain that is common in several +TIPs and regulates initiation of retrograde transport in neuronal cells^36^,^37^. Interestingly, mammalian TBCB, another member of tubulin-folding cofactor family, also has the CAP-Gly domain and interacts with p150^Glued,^ 38. +TIPs are a structurally and functionally diverse group of proteins that are distinguished by their specific accumulation at microtubule plus ends, or growing ends^39^,^40^. +TIPs control different aspects of microtubule dynamics and form links between microtubule ends and other cellular structures^2^. Some of +TIPs also participate in microtubule-actin crosstalk, such as CLIP-170–formin interaction and EB1-RhoGEF2 interaction^2^. There is accumulating evidence that Strip and also other STRIPAK components (Mst3, Mst4, and Ccm3) regulate the actin network^9^,^13^. For example, Strip1 and 2, homologs of *Drosophila* Strip, were recently identified as regulators of the actomyosin contraction that regulates cell migration in cancer cells^41^. Taken together, Strip and STRIPAK seem to form a giant complex with TBCD and Glued at growing ends of microtubule to stabilize them and serve as a linker between microtubules and actin networks to regulate proper neurite branching and elongation.

*strip* knockdown resulted in a decrease in the level of acetylated *α*-tubulin. Therefore, we examined the possibility that Strip directly affects the acetylated tubulin level by regulating acetylation or deacetylation enzymes. We investigated the genetic interaction between *strip* and *HDAC6*, one of the enzymes responsible for the deacetylation of *α*-tubulin^42^, however, the shorter axon phenotype of *strip* knockdown was not suppressed when combined with a *HDAC6* mutation (data not shown). Thus, Strip does not seem to directly affect the acetylation of *α*-tubulin, but affect the stabilization of microtubule by interacting with TBCD, Glued, and other molecules. Although controversial, some reports indicate that acetylation is the result, and not the cause, of stabilization^19^,^43^,^44^.

Many diseases are linked to microtubules. For example, defects in retrograde transports along microtubules cause neurodegenerative diseases, such as motor neuropathy 7B^45^ and Perry syndrome^46^. Furthermore, Dscam is implicated in the cognitive disabilities in Down syndrome. Moreover, STRIPAK complexes have been linked to a number of clinical conditions and diseases^9^. Cancer genome sequencing also identified frequent mutations in human *STRIP2*, and based on the mutation frequency and types, *STRIP2* was classified as an oncogene^47^. Thus, further investigation of Strip, STRIPAK, and the microtubule complex would yield new insights into the mechanisms of various diseases.

## Methods

### Fly strains

Flies were maintained under standard laboratory condition (25 °C). *UAS-Dscam17.2-GFP*^29^ was a kind gift from Tzumin Lee. We previously generated *strip*^*dogi* 14^. *UAS-TBCD* used in previous study^16^ is different from the one we used in this report. They are independent transgenic lines generated from the same construct^16^. The former one is inserted in third chromosome, and the one we use in this study is on second chromosome (*UAS-TBCD*^*weak*^). To generate *UAS-shRNA-TBCD*^*#1*^, we used the protocol previously described^48^. The target sequence of the *shRNA-TBCD*^*#1*^ is 5′- GGAGCTGAATGAACTAATAATA -3′, which is different from the *shRNA* we used in the previous study^16^. The fragment was subcloned into the *pUAST-attB* vector. Transgenic flies were raised by BestGene.

### Clonal analysis

We used the MARCM method^49^. Briefly, we crossed MARCM-ready flies that contain *FLP* recombinase, an *FRT* site, *GAL4*, tubulin 1α promoter-*GAL80*, and *UAS-mCD8GFP* to a line containing the corresponding *FRT* and mutation of interest for MARCM analysis. To analyze DL1 PNs, we used the *GH146-Gal4* driver and heat-shocked flies (37 °C, 1 h) at 0–24 h after larval hatching (ALH). The anterodorsal PN single-cell clones generated during this time period innervated the DL1 glomerulus. The penetrance of overbranching phenotype of *strip*^*dogi*^PNs was considerably reduced in this study compared to the previous report^14^. This was because of the difference in the heat-shock timing. In the previous study^14^, we heat-shocked flies 0–30 h ALH for the phenotype analysis of *strip*^*dogi*^PNs. To analyze mushroom body *α*/*β* neurons, we used the *OK107-Gal4* driver and heat-shocked flies (37 °C, 1h) at 0–24 h after puparium formation. Dissections were performed on both sexes of adults aged 1–10 days.

### Immunohistochemistry

The fixation, immunostaining, and imaging of the fly brains were carried out as previously described^50^. Briefly, the brains were dissected in 0.3% Triton X-100 (vol/vol) in phosphate-buffer (PBT), followed by fixing with 4% paraformaldehyde (wt/vol) in PBT at room temperature (RT) for 20 min, and subsequently blocked in PBT with 5% normal goat serum (vol/vol) for 1 h at RT. Primary and secondary antibody incubations were carried out in PBT for overnight at 4 °C.

For *Drosophila* S2 cells, cells were cultured on concanavalin A-coated coverslips, fixed, and immunostained as previously described^51^. Briefly, S2 cells were grown for 1 h at 26 °C on concanavalin A-coated coverslips, and were fixed with 4% paraformaldehyde (wt/vol) in 0.3% Triton X-100 (vol/vol) in phosphate-buffer saline (PBST) at RT for 10 min, and subsequently blocked in PBST with 5% normal goat serum (vol/vol) for 30 min. For investigating the colocalization of Strip and TBCD, we transfected S2 cells with *Gal4-actin promoter* and *pUAST-TBCD-myc*^16^ by utilizing Effectene Transfection Reagent (Qiagen) and cultured for 24 h at 26 °C before plating on concanavalin A-coated coverslips. For clear observation of the microtubules, we performed the extraction method^17^. We rinsed S2 cells cultured on concanavalin A-coated coverslips with PEM buffer (100 mM PIPES[pH 6.9], 1 mM EGTA, 1 mM MgSO_4_) once, and treated them with extraction buffer (100 mM PIPES [pH 6.9], 1 mM EGTA, 1 mM MgSO_4_, 1% Triton X-100, 2% paraformaldehyde, 10 μM taxol). After incubation for 4 min at RT, the cells were fixed with 4% paraformaldehyde (wt/vol) in PEM buffer for 20 min at RT and subsequently blocked in PBST with 5% normal goat serum (vol/vol) for 30 min. Primary and secondary antibody incubations were carried out in blocking solution for 1 h at RT. We used the following antibodies: anti-Strip^14^ (rat, 1:50), anti-*α*-tubulin (mouse, 1:2000, Sigma T6199), anti-acetylated-*α*-tubulin (mouse, 1:1000, Sigma T7451), anti-EB1^52^ (rabbit, 1:1000, a kind gift from S. L. Rogers), anti-myc (mouse, 1:1000, Invitrogen), anti-Brp (mouse, 1:40, DSHB nc82), anti-mCD8 (rat, 1:200, Invitrogen), anti-FasII (mouse, 1:40, DSHB 1D4) antibodies. For the anti-acetylated-*α*-tubulin staining in S2 cells, we co-stained cells with anti-*α*-tubulin (mouse) and anti-acetylated-*α*-tubulin (mouse) antibodies by utilizing the Zenon labelling technology. S2 cells were incubated with anti-acetylated-*α*-tubulin for 1 h and subsequently incubated with anti-mouse IgG conjugated with Alexa Flour 488 for 1 h. Lastly, S2 cells were cultured with anti-*α*-tubulin that was conjugated with Alexa Flour 647 beforehand by the Zenon Alexa Flour 647 Mouse IgG_1_ labeling kit (life, Z-25008).

### dsRNA generation and treatment

The dsRNA design, production, and treatment were performed as follows^51^. Briefly, 700–900 bp gene-specific sequences with T7 RNA polymerase sequence at both ends were amplified by PCR, and *in vitro* transcription was performed using T7 RiboMAX Express RNAi System (Promega) according to the manufacturer’s protocol. For *strip* dsRNA, templates for *in vitro* transcription were generated using the primers 5′-TAATACGACTCACTATAGGGCCTGCATAAACCTGCTGCGC-3′ and 5′-TAATACGACTCACTATAGGGCTAGAGGGCGTCCCAGTCGG-3′. For *TBCD* dsRNA, templates for *in vitro* transcription were generated using the primers 5′-TAATACGACTCACTATAGGGGTGGTTTACCTCTCCAACCAACGG-3′ and 5′-TAATACGACTCACTATAGGGCTGTATGCCTGGATGTTCTCGCGG-3′. For control dsRNA, primer sequences were used to amplify the sequence from the bacterial cloning plasmid pBluescript SK^51^. For Fig. 2, *Drosophila* S2 cells (1.0 × 10^6^) were cultured in a 35-mm dish; 30 μg control or *strip* dsRNA was applied every 2 days for 8 days. For Fig. 3c, *Drosophila* S2 cells (1.0 × 10^7^) were cultured in a 100-mm dish; 120 μg control or *TBCD* dsRNA was applied every 2 days for 6 days.

### Immunoprecipitation

*Gal4-actin promoter* and desired UAS construct (*UAS-TBCD-myc*, *UAS-strip* or *UAS-Dscam17.1GFP*) were co-transfected with Effectene Transfection Reagent (Qiagen); 6.0 × 10^6^ *Drosophila* S2 cells per 60-mm dish were plated, and transfection was performed according to the manufacturer’s protocol. After 48 h in culture, S2 cells were collected, sonicated in the lysis buffer (25 mM Tris-HCl, pH 7.9, 10 mM NaCl, 2 mM EDTA, 0.5% Triton X-100, 10 mM DTT, and 1 x cOmplete (cocktail of protease inhibitors, Roche)), and incubated with anti-Strip antibody (rat, 1:100), control IgG, or anti-GFP (rabbit, 1:500, MBL) overnight. Protein G agarose (Roche) was added, and immunoprecipitation was performed according to the manufacturer’s protocol.

### Immunoblotting

Western blotting was performed according to standard techniques. Briefly, we subjected 2–10 μg of S2 cell lysates to SDS-PAGE analysis (12.5% for Histone H3; for others, 7.5% or 10%) and immunoblotting. The following antibodies were used for immunoblotting: the anti-Strip antibody^14^ (rabbit, 1:200), the anti-Strip antibody^14^ (rat, 1:50), the anti-*α*-tubulin (mouse, 1:2000, Sigma T6199), the anti-acetylated-*α*-tubulin (mouse, 1:1000, Sigma T7451), the anti-tyrosinated-*α*-tubulin (mouse, 1:1000, Sigma T9028), the anti-Histone H3 antibody (rabbit, 1:2000, Active motif 39163) and the anti-TBCD antibody^16^ (guinea pig, 1:1000). All data are representative of more than three biological replicates.

### Microtubule co-sedimentation assay

This assay was carried out as previously described^53^. Porcine tubulin was polymerized in a tubulin buffer (40 mM PIPES pH6.9, 1 mM MgCl_2_, 1 mM EGTA, 1 mM GTP, 1 mM DTT) by adding Taxol (20 μM) for 20 min at RT. *Drosophila* S2 cells from two 100-mm confluent dishes were collected, rinsed with PBS, re-suspended in a buffer (40 mM PIPES pH6.9, 1 mM MgCl_2_, 1 mM EGTA, 1 mM DTT, 1% NP40, 20μM Latrunculin B with protease inhibitors), and centrifuged at 156,500 g for 20 min. The supernatant of this S2 extract was split in half, incubated either with *in vivo*-polymerized microtubules or buffer only for 30 min at RT and pelleted through 30% glycerol cushion (30% glycerol, 40 mM PIPES pH6.9, 1 mM MgCl_2,_ 1 mM EGTA, protease inhibitors, 20 μM Taxol only for the vial that contains microtubule) by centrifugation at 194,000 g for 90 min at 4 °C. The pellet was collected and separated by SDS-PAGE and analyzed by western blotting.

### Image acquisition

For all experiments, images were obtained using TCS-SP5 confocal laser scanning confocal microscopy (Leica) with a PL APO CS 40×/1.25 Oil lens (Leica) for PN and mushroom body neuron imaging or a PL APO CS 63x/1.40 Oil lens (Leica) for imaging S2 cells. Fields of view were randomly selected and each biologically independent experiment was repeated more than two times.

### Image analysis

For Fig. 2a–d, all images were blinded prior to classification to avoid experimental bias. The investigator who conducted the blind test was different from the investigator who performed the experiments.

### Statistical analysis

Statistical analysis was performed using Prism 5 software (GraphPad). The chi-square test was used to compare data variations between control and *strip* dsRNA-treated S2 cells and data variations of phenotypes of mushroom *α* and *β* lobes.

## Additional Information

**How to cite this article**: Sakuma, C. *et al*. A STRIPAK component Strip regulates neuronal morphogenesis by affecting microtubule stability. *Sci. Rep*. **5**, 17769; doi: 10.1038/srep17769 (2015).
